# Artificial Intelligence in the Design and Optimization of Orthodontic Materials: A Clinical Perspective on Current State and Future Directions

**DOI:** 10.3390/ma19122538

**Published:** 2026-06-12

**Authors:** Marcin Mikulewicz, Anna Paradowska-Stolarz

**Affiliations:** Department of Dentofacial Orthopedics and Orthodontics, Division of Facial Abnormalities, Medical University of Wroclaw, 50-425 Wroclaw, Poland; anna.paradowska-stolarz@umw.edu.pl

**Keywords:** artificial intelligence, machine learning, orthodontic materials, clear aligners, NiTi archwires, additive manufacturing, periodontal ligament, biomaterials, digital orthodontics, deep learning

## Abstract

Artificial intelligence (AI) has transformed orthodontic diagnosis, yet its application to orthodontic materials science remains critically underexplored. This perspective identifies and characterizes the AI–materials integration gap as the central unresolved problem in digital orthodontics: AI-optimized treatment plans are currently executed through empirically selected materials whose mechanical behavior is never modeled by the planning system. We examine four domains where this gap is consequential: thermoplastic aligner polymers (PETG vs. TPU), where supervised ANNs can predict force decay from polymer composition; NiTi archwire alloys, where Bayesian optimization and Gaussian process regression are accelerating alloy design; additive manufacturing of orthodontic devices, where supervised ML reduced print-parameter optimization burden in a 2025 five-variable surface roughness study; and AI-driven biological response prediction, where FEA-surrogate neural networks reduced biomechanical computation from minutes to milliseconds per patient query. A scoping review of clear aligner AI identified 41 studies—none addressing aligner material properties as a primary outcome. We argue that closing the AI–materials gap requires standardized open material-performance datasets; FEA-surrogate models integrating polymer stiffness as a treatment-planning input; patient-specific digital twins with defined material, mechanical, and biological parameter layers; and federated learning infrastructure spanning clinics and manufacturers.

## 1. Introduction

The past decade has witnessed an accelerating convergence of artificial intelligence (AI) and clinical orthodontics. Machine learning algorithms now automate cephalometric landmark detection with sub-millimeter precision, convolutional neural networks (CNNs) classify skeletal malocclusions from lateral cephalograms with accuracy comparable to experienced clinicians, and remote monitoring systems use AI to track tooth movement between appointments [1,2,3]. The volume of published literature in this domain has grown year on year, and multiple comprehensive reviews have mapped AI applications across diagnosis, treatment planning, and patient monitoring [4,5,6].

For the purposes of this perspective, we define materials optimization as encompassing three distinct decision layers: (1) composition—the selection or design of the constituent monomers, polymer blends, or alloying elements that determine the intrinsic mechanical and biological properties of the material; (2) processing—the manufacturing parameters (thermoforming temperature, print layer thickness, heat treatment protocol) that translate a given composition into a finished device with specific in-use properties; and (3) selection—the patient-specific choice of which available material to deploy for a given clinical scenario, given the patient’s anatomy, biomechanical requirements, and biological risk profile. These three layers are methodologically distinct: composition optimization addresses the design space, processing optimization addresses the manufacturing space, and selection optimization addresses the clinical decision space. AI tools relevant to each layer are different, and conflating them obscures both the current state of the field and the priorities for future research.

Yet, a fundamental asymmetry persists, and its dimensions can be quantified. A 2024 scoping review of AI in orthodontic diagnosis and treatment planning—the most comprehensive to date, covering 71 studies—classified the entire field into three domains: diagnostics (*n* = 29), landmark identification (*n* = 20), and treatment planning (*n* = 22); no study in the corpus addressed orthodontic material properties as a primary outcome [7]. A bibliometric analysis of 1368 AI-in-dentistry studies published through 2024 confirmed that classification, detection, and segmentation tasks—all diagnostic in nature—dominate the literature, while material design and property optimization are not represented as a recognized research category [8]. These structural omissions are not accidental: they reflect a field that has organized itself around the diagnostic image as the primary data object, treating material selection as a downstream clinical decision outside the AI loop. While AI has been applied extensively to the question of where teeth should move and whether movement is proceeding as planned, the question of what material should be used to generate that movement—and how AI might optimize it—has received strikingly little systematic attention. The force a tooth receives is determined not by the diagnostic algorithm but by the physical properties of the aligner polymer, archwire alloy, or bracket material through which the plan is executed. In the authors’ clinical experience, material selection in daily orthodontic practice remains largely empirical: polymer grades and wire alloys are chosen from habit, manufacturer claims, or broad clinical guidelines, rarely from quantitative material-matching to individual patient biomechanics—a characterization consistent with the broader observation that contemporary orthodontic decision-making remains largely phenotype-driven, with individualized biological and biomechanical attributes not yet integrated into routine clinical decision protocols [9].

The contrast with adjacent fields is instructive and sharpens the urgency of this gap. In dental biomaterials research, more broadly, AI-driven material optimization is already advancing: Gaussian process regression combined with Bayesian optimization has recently been applied to predict and optimize the viscosity of dental resin composites across formulation space, identifying optimal filler compositions within seven experimental iterations—a sample efficiency that manual testing cannot approach [10]. In regenerative dentistry, generative AI models are being used for inverse material design, in which a target biological property is specified, and the algorithm navigates composition space to propose candidate materials that satisfy it [11]. Orthodontics—a discipline whose clinical outcomes are as directly determined by material behavior as any dental specialty—has not yet participated in this transition. The present perspective argues that this lag is consequential and correctable and identifies the specific research agenda required to close it.

**Figure 1:**
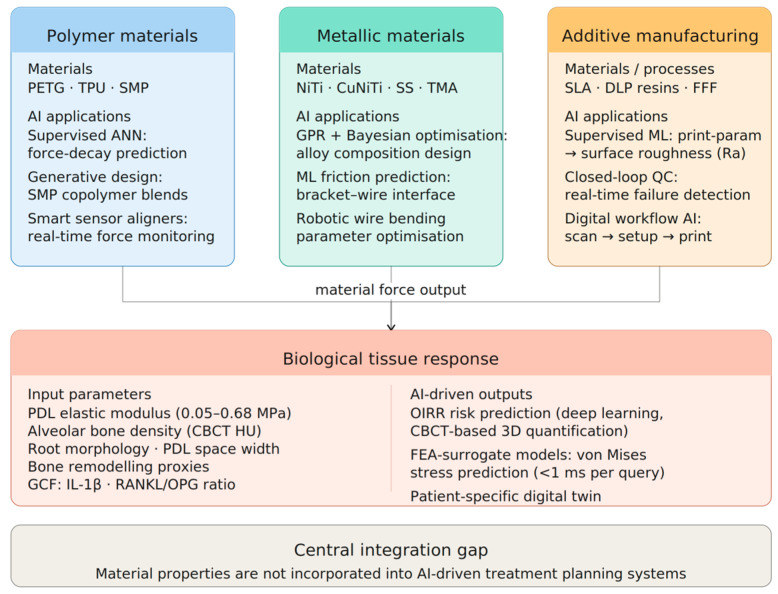
Schematic of the four AI–material integration domains in orthodontics examined in this perspective and the central integration gap: material properties are currently not incorporated into AI-driven treatment planning systems. PDL = periodontal ligament; OIRR = orthodontically induced root resorption; SLA = stereolithography; DLP = digital light processing; NiTi = nickel-titanium; SMP = shape memory polymer; ANN = artificial neural network; QC = quality control.

This perspective argues that the most consequential near-term contribution of AI to orthodontics will not be further refinement of diagnostic image analysis—a field approaching performance ceilings on well-defined tasks—but rather the rationalization of material selection, acceleration of material development, and patient-specific prediction of biological responses to material-generated forces. Purely diagnostic AI in orthodontics has been comprehensively reviewed elsewhere [12]. The aim of this perspective is to examine four interconnected material domains—thermoplastic aligner polymers, metallic archwire, and bracket alloys, additive manufacturing of orthodontic devices, and AI-assisted modeling of periodontal and bone tissue responses—mapping the current state of AI application in each, identifying the specific gaps that limit clinical utility, and outlining the research directions that would close them. Literature is drawn from 2022 to 2026. Figure 1 illustrates the four AI–material integration domains and the central integration gap addressed in this perspective.

## 2. AI in Thermoplastic Aligner Material Development

Clear aligner therapy (CAT) is the fastest-growing modality in contemporary orthodontics, with over 17 million patients treated globally by 2023 [13]. The dominant aligner materials—polyethylene terephthalate glycol (PETG) and thermoplastic polyurethane (TPU)—differ substantially in their mechanical behavior, and these differences have direct clinical consequences. PETG aligners exhibit higher stiffness and tend to generate greater orthodontic forces, producing more root movement, while TPU produces predominantly crown movement with reduced force decay over the wear cycle [14]. Despite this, material choice in clinical practice is made empirically, without quantitative modeling of how a given polymer’s properties will interact with a specific patient’s periodontal tissue stiffness and root morphology.

First, supervised machine learning models—specifically feedforward ANNs trained on labeled polymer-property datasets—are being developed to predict force delivery and stress relaxation from polymer composition and processing parameters, enabling in silico material screening before physical fabrication. ANNs have demonstrated utility in modeling the thermoforming process, predicting how parameters such as forming temperature, sheet thickness, and cooling rate affect the final mechanical properties of the aligner [15]. Second, embedded sensor systems are beginning to generate the real-world material performance data that training such models requires. A landmark 2025 study by Feng et al. developed the ARIA system—a transparent aligner with high-performance piezoelectric sensors embedded into the occlusal surface via flexible printed circuits—which achieved 95% accuracy in malocclusion type classification from over 1400 force datasets and enabled real-time wireless monitoring of bite force distribution across eight dental sites [16]. This represents not merely a diagnostic tool but a material-integrated measurement platform: force data collected by such systems could directly train ML models linking polymer stiffness parameters to in vivo force delivery.

The clinical relevance of PETG vs. TPU selection becomes concrete when managing specific movement types. In the authors’ clinical practice, a recurring scenario illustrates this gap precisely: patients undergoing bodily retraction of maxillary incisors—a movement that demands controlled root torque—have shown markedly different outcomes depending on aligner polymer. Cases managed with PETG-based aligners consistently achieved greater root apex displacement, consistent with the higher stiffness and root-force generation documented for this material [14]. In contrast, TPU-based aligners in analogous cases produced predominantly crown tipping, requiring attachment redesign to compensate. This is not a failure of the AI-generated virtual setup, which was identical in geometric prescription; it is a failure of the material to execute the intended biomechanical prescription. No current AI system would have predicted or prevented this outcome, because no AI system integrates polymer mechanical properties into its force prediction. The clinical observation described here directly motivates the research agenda for AI-driven material selection: a system that ingests patient-specific PDL compliance, root morphology, and treatment-plan movement type and outputs the optimal polymer grade and aligner thickness does not yet exist but is technically achievable within the current state of ML methodology. These observations are consistent with published biomechanical data [14] but represent expert clinical experience rather than prospectively controlled comparative data.

Data needs and speculated use case. Predicting aligner polymer forces with ML model development involves “training” the models with three different types of data collected in a coordinated manner: (a) in vitro mechanical testing data like stress relaxation curves, elastic modulus, and dynamic mechanical analysis curves collected under oral conditions (temperature of 37 °C, along with sheet thickness) for each polymer grade; (b) in vivo force measures from a sensor-fitted aligner like the ARIA system (target data); and (c) treatment results (rate and trajectory of actual tooth movement with respect to the virtual setup). Training models without including effects due to attachment design and placement, staging (sequential vs. simultaneous tooth movements), aligner fit during insertion, and patient compliance (wear time) would lead to unreliable force predictions beyond the given training scenarios. The conceived clinical application of the ML model may be as follows: upon initial treatment planning phase, the clinician could input patient-specific data from the cone beam computed tomography (CBCT) scan, along with the tooth movement type and other treatment factors, into the model; the model then outputs a recommended polymer grade and sheet thickness ranked by the predicted range of forces it could generate (along with model uncertainty). During the course of treatment, the model could be re-evaluated for predicted forces or tooth movement. The objective of the ML model is to serve as a clinical advisor rather than autonomously deciding the material selection (clinician remains the final judge).

Beyond PETG and TPU, the next wave of aligner materials is already emerging. Shape memory polymers (SMPs)—materials that recover from a temporary deformed shape to a stable programmed shape under thermal or mechanical stimulus—offer the theoretical prospect of aligners that self-activate at body temperature, eliminating the compliance dependency that currently limits CAT outcomes [14]. AI-driven generative design for SMP composition and geometry represents a nascent but high-potential research direction: reinforcement learning models could optimize the balance between shape recovery force, biocompatibility, and optical transparency simultaneously. Copolymer blending strategies, including polycarbonate as a compatibilizer between PETG and TPU to tune stress relaxation profiles, represent another target for generative ML-guided material design, in which multi-objective optimization algorithms search copolymer composition space under biocompatibility and mechanical constraints [14].

A scoping review of AI in clear aligner therapy identified 41 relevant studies, with the largest clusters in tooth segmentation (*n* = 16), digital setup (*n* = 13), and remote monitoring (*n* = 8), while AI applied to aligner material properties per se was not represented as a distinct category [13]. This gap confirms the clinical material disconnect: the same digital workflow that generates a precise virtual setup currently makes no use of the physical properties of the polymer through which that setup will be executed. Closing this gap—by incorporating material stiffness, force decay curves, and patient-specific periodontal compliance into AI-driven aligner design—is, in the present authors’ view, the single highest-yield research target in the field.

## 3. AI in Orthodontic Archwire and Bracket Material Science

Fixed orthodontic appliances remain the most widely used treatment modality globally, and their performance is governed by the mechanical properties of archwire alloys and the tribological characteristics of the bracket–wire interface. Nickel-titanium (NiTi) archwires are the dominant choice for the alignment and leveling phase of treatment due to their superelastic behavior—a property arising from a reversible stress-induced martensitic phase transformation that enables large deformations to be recovered while delivering a near-constant unloading force. This characteristic is ideal for initial tooth alignment, but NiTi’s superelastic plateau force is sensitive to alloy composition, wire diameter, heat treatment history, and oral temperature variation, making in vivo force prediction from standard in vitro tests unreliable [17].

Machine learning is increasingly applied to NiTi and shape memory alloy design in the broader materials science literature, with direct methodological relevance to orthodontics. A 2025 review of ML approaches across alloy systems by Rahman et al. demonstrated that adaptive design strategies—combining ML predictions with targeted experiments—can accelerate the identification of NiTi-based alloy compositions with specified transformation temperatures and superelastic behavior, substantially reducing the experimental burden of alloy development [18]. The dominant ML paradigms in alloy design are Gaussian process regression (GPR) and Bayesian optimization for sample-efficient experimental design and graph neural networks (GNNs) for property prediction from alloy composition graphs. GPR is particularly suited to the small-dataset regime typical of orthodontic in vitro studies as it provides calibrated uncertainty estimates that guide targeted experimental sampling rather than requiring exhaustive combinatorial testing. These methods, widely adopted in aerospace and biomedical stent research, have not yet been systematically applied to orthodontic wire alloy optimization. The specific clinical targets—controlled unloading force magnitude, minimal hysteresis, resistance to fatigue under oral conditions, and biocompatibility—constitute a well-defined multi-objective optimization problem well suited to ML-guided alloy design.

At the bracket–wire interface, friction governs the efficiency of sliding mechanics and directly determines the net force available for tooth movement. The predictive variables required for an ML-based friction prediction model fall into three categories: (a) material descriptors—wire alloy composition, bracket material (stainless steel, ceramic, polymer-composite), wire surface roughness (Ra) measured under ISO 21920-2 [19], and bracket slot surface finish; (b) geometric descriptors—wire cross-section, slot dimensions, and second- and third-order angulation between wire and slot; and (c) interface descriptors—ligation type (elastomeric, metal, self-ligating active or passive), saliva or artificial saliva contamination, and applied normal force. Stainless steel archwires demonstrate the lowest surface roughness (mean Ra approximately 0.25 μm) and lowest frictional resistance among conventional materials, while esthetic archwires show the highest roughness (mean Ra approximately 0.40 μm) and correspondingly elevated friction [20]. Standardization of friction measurement is currently inadequate for ML training purposes: published friction studies use widely varying test geometries, sliding velocities, and lubrication conditions, which prevents pooling data across studies. A consensus measurement protocol—specifying test temperature (37 °C), medium (artificial saliva), sliding velocity (≤0.5 mm/min), and normal force range—is a prerequisite for the inter-laboratory data pooling that ML model training requires. ML models trained on such standardized multi-parameter datasets could generate patient-specific friction predictions that inform wire sequencing decisions currently made by clinical judgment alone.

Looking forward, AI is beginning to bridge the gap between three-dimensional treatment planning and physical appliance fabrication. Algorithms that translate a virtual setup into precise archwire bending instructions—analyzing desired torque, tip, and inter-arch relationships to predict optimal curvature for each wire segment—represent a direct application of AI to material forming [21]. This digital-to-physical translation, currently implemented in robotic wire bending systems, creates a data stream of bending parameters, material responses, and clinical outcomes that, once systematically collected, could train predictive models of the optimal wire sequence for a given case. In clinical orthodontic practice, wire sequencing decisions—the choice of initial alignment wire, the progression to working archwires, and the selection of finishing wires—remain among the least evidence-based choices in routine fixed appliance therapy. A systematic review and meta-analysis of 16 randomized controlled trials covering 1108 patients concluded that current evidence is insufficient to support recommendations for the majority of initial archwires or for any specific archwire sequence [22].

## 4. AI-Driven Additive Manufacturing of Orthodontic Devices

Three-dimensional printing has transitioned from a laboratory novelty to a clinical production technology in orthodontics. Patient-specific aligners, indirect bonding trays, retainers, and surgical guides are now routinely fabricated by additive manufacturing, predominantly by stereolithography (SLA) and digital light processing (DLP) using photopolymer resins. The material question in this context is not which polymer to select from a catalog but how to optimize the printing process—layer thickness, exposure time, print orientation, post-cure duration—to achieve specific mechanical targets in the final device [23].

It is essential to distinguish two distinct additive manufacturing use cases in orthodontics, which carry different regulatory and material-performance requirements. The first is indirect manufacturing: 3D-printed models, aligner thermoforming molds, and surgical guides that are not in direct prolonged patient contact and whose mechanical performance is judged primarily by dimensional accuracy. The second is direct manufacturing of patient-contact devices: 3D-printed aligners, indirect bonding trays, and retainers fabricated from biocompatible photopolymer resins, which contact mucosa for extended periods. The AI optimization questions, regulatory pathways, and safety endpoints differ substantially between the two. This perspective addresses both but distinguishes them where the discussion is use-case specific.

Supervised machine learning—in which models are trained on labeled datasets of print parameter inputs and measured surface quality outputs—has demonstrated direct utility in this optimization problem. A 2025 study applied artificial neural networks (ANNs), support vector regression (SVR), and related approaches to predict surface roughness of 3D-printed dental resin devices from five input parameters—layer thickness, infill density, print angle, exposure time, and lift speed—demonstrating that ML models can guide print parameter selection toward specific surface quality targets without requiring extensive physical trial and error [23]. Surface roughness is one of several clinically significant endpoints that AI-driven process optimization must address. Surface roughness affects plaque accumulation on aligners and retainers (caries and periodontal risk) and bracket-adhesive interface bond strength. However, the full set of safety-relevant material endpoints for additively manufactured patient-contact devices also includes the following: mechanical strength under cyclic loading (flexural and tensile fatigue resistance, relevant to clinical fracture risk); creep behavior under sustained orthodontic-relevant loads (relevant to force decay over wear periods); water sorption and solubility (per ISO 4049 [24]), which modulate dimensional stability and surface degradation in the oral environment; and residual monomer release (measurable by HPLC), which is the principal biocompatibility concern for photopolymer resins. ML models for AM process optimization must ultimately predict all of these endpoints, not surface roughness alone. The 2025 study cited above [23] represents an important proof of concept for ML-based process optimization but addresses only one of these endpoints; equivalent studies for mechanical fatigue, creep, water sorption, and monomer release are conspicuously absent from the current literature.

The integration of AI across the full digital workflow—from intraoral scan through AI-assisted tooth segmentation, automated virtual setup, generative aligner geometry design, and optimized 3D printing—is creating a closed-loop manufacturing system in which AI governs both the geometric prescription and the material process parameters [13]. This convergence is further extended by AI-integrated quality control: closed-loop 3D printing systems have demonstrated waste reduction of up to 90% through real-time detection of print failures and automated reuse of unprocessed resin, without compromising dimensional accuracy [25].

However, significant material science gaps limit the clinical reliability of additively manufactured orthodontic devices. Long-term mechanical performance data for 3D-printed orthodontic resins under oral conditions—cyclic loading, thermal variation, salivary exposure—remain substantially thinner than for thermoformed thermoplastics. Biocompatibility data sufficient to support ML model training for material selection are fragmented and largely proprietary. Regulatory frameworks for AI-designed and AI-process-optimized patient-contact devices do not currently exist in a coherent form under either FDA SaMD guidance or the European MDR. In the authors’ assessment—based on the disparity between the rapid commercial diffusion of orthodontic 3D printing and the comparatively limited published evidence on long-term mechanical and biological performance of patient-contact printed devices—additive manufacturing in orthodontics has advanced faster than the material characterization and regulatory infrastructure required to support its safe, evidence-based clinical use. We do not attempt to quantify this lag but consider it the central translational risk of the domain.

## 5. AI for Predicting Biological Responses to Orthodontic Material-Force Interactions

The most clinically consequential—and scientifically underdeveloped—application of AI in orthodontic materials science is the prediction of biological tissue responses as a function of the forces generated by specific materials. Orthodontic tooth movement is mediated by the periodontal ligament (PDL), a viscoelastic connective tissue whose mechanical properties determine how applied forces are transmitted to alveolar bone and root cementum. These biological material properties are not currently integrated into AI-driven orthodontic planning systems, which treat tissue response as an unmodeled biological variable—a population-level uncertainty applied uniformly across patients rather than a patient-specific input parameter.

Orthodontically induced root resorption (OIRR) affects 40–60% of orthodontic patients to some degree and represents the most significant irreversible adverse effect of orthodontic treatment [26]. AI has made substantial advances in detecting and quantifying OIRR from CBCT images after it has occurred. A 2025 study by Zheng et al. developed a deep learning model for automatic 3D quantification of OIRR from CBCT, achieving strong consistency with manual measurements while reducing processing time from 5–7 h per patient to 20–30 min [26]. An independent validation study using AI-aided segmentation confirmed high specificity (100%) for apical third resorption detection [27]. AI-driven root and bone predictions have also been proposed as a direct aid to clear aligner treatment planning, enabling pre-treatment assessment of resorption risk prior to force application [28]. The PDL, alveolar bone, and root cementum are themselves biological materials with quantifiable properties: PDL elastic modulus (approximately 0.05–0.68 MPa depending on loading direction and rate, though published values span several orders of magnitude depending on experimental methodology [29]), bone density measurable from CBCT, and cementum susceptibility to resorption dependent on root morphology and genetic factors [30]. Understanding these tissue material properties is essential context for the prospective risk prediction models.

A note on bone density values derived from CBCT is in order. In contrast to traditional medical CT, there is no standard absolute Hounsfield unit (HU) calibration across different scanner manufacturers, voxel sizes, or field-of-view dimensions in CBCT. HU values for the same region can vary significantly across CBCT scanners, and the values can fluctuate for a given scanner as well. When ML algorithms use bone density derived from CBCT as a feature for prediction, this non-standard calibration can lead to data inconsistency across institutions, limiting the generalizability of the ML algorithms. Relative measures of density such as ratios across regions within the same scan are more reliable than absolute values, and novel calibration approaches employing phantoms can improve the situation. However, until density values from CBCT are standardized across scanners, ML algorithms can instead account for the calibration uncertainties by including scanner type and acquisition parameters in the algorithm, rather than using CBCT HU values as the absolute values of bone density. Polymer grade and sheet thickness are predicted to deliver the prescribed force within an acceptable range, along with a calibrated uncertainty estimate. During treatment, periodic re-evaluation against measured force or tooth movement updates the prediction. This represents a clinical decision support tool, not an autonomous prescription system: final material selection remains with the clinician.

AI-enhanced CBCT segmentation has further demonstrated accurate volumetric quantification of resorption across treatment stages [31]. A subsequent analysis integrating deep-learning-based segmentation with volumetric risk factor modeling found that treatment duration, root morphology (pipette-shaped roots), and applied force level were independently associated with OIRR severity—with force level, critically, being a direct function of the material used [30]. These tools represent significant diagnostic advances. However, they detect damage retrospectively—they do not predict, prior to treatment, which patients are at elevated risk given their specific PDL material properties and the force magnitudes that a given aligner polymer or archwire will generate.

AI-driven biomechanical modeling offers a pathway toward prospective risk prediction. Finite element analysis (FEA) of PDL and alveolar bone stress under orthodontic loading has established the theoretical framework linking force magnitude, distribution, and direction to tissue response. The computational cost of patient-specific FEA, however, has prevented real-time clinical application. A 2025 proof-of-concept study demonstrated that feedforward neural networks trained on FEA-derived datasets of dental implant loading scenarios—varying load intensity, angle, and implant geometry across 200 simulations—could predict von Mises stress, displacement, and fatigue safety factors with high fidelity, reducing computation time from minutes to milliseconds per query [32]. This FEA-surrogate approach is directly applicable to orthodontic PDL modeling: neural networks trained on parametric libraries of FEA simulations spanning root morphology, PDL thickness, bone density, and applied force magnitude could enable patient-specific biomechanical risk assessment at the point of treatment planning, without the computational burden of solving a new finite element system for each patient. An alternative and complementary approach uses physics-informed neural networks (PINNs), which embed the partial differential equations governing tissue mechanics directly into the network’s loss function. Unlike purely data-driven surrogates, PINNs can generalize to anatomical configurations outside the training distribution—a critical advantage given the morphological variability of root and PDL geometry across patients. PINNs have not yet been applied to orthodontic PDL modeling but represent a methodologically appropriate next step beyond FEA-surrogate approaches.

Critically, such surrogate models could incorporate material parameters—the stiffness of the specific aligner polymer being used, the unloading force plateau of the selected NiTi wire—as input variables, directly linking material choice to predicted tissue response. A 2025 review identified that combining AI with biomechanical simulations could lead to superior force distribution models, minimizing risks such as root resorption, and that advances in dental materials enhanced by AI-driven design could yield more effective and durable orthodontic appliances [3]. The key word is combining: no existing clinical AI system integrates material stiffness as an input variable in biological response prediction. The material is still selected outside the AI loop. Closing this integration gap—by treating both the biological tissue and the orthodontic appliance as materials with quantifiable properties that interact in a predictable, learnable relationship—is the research agenda the present authors call for explicitly. Table 1 summarizes the AI applications, identified gaps, and clinical relevance across the four orthodontic material domains examined in Section 2, Section 3, Section 4 and Section 5.

## 6. Limitations and Open Problems

### 6.1. Data Scarcity and Non-Standardization

Material performance datasets for orthodontic applications are fragmented, largely proprietary, and methodologically heterogeneous. In vitro mechanical testing of aligner polymers is conducted under varying temperature, humidity, and loading conditions across studies, preventing meta-analytic synthesis. No open benchmark dataset equivalent to public cephalometric databases exists for aligner polymer or archwire alloy performance under defined oral conditions. This is the primary bottleneck for ML model development in orthodontic materials science: algorithms require large, standardized, labeled datasets, and the orthodontic materials literature does not yet provide them. The absence of standardized reporting—analogous to MI-CLAIM in clinical AI or CONSORT in clinical trials—for material AI studies further limits reproducibility.

To the authors’ knowledge, no dedicated open repository, benchmark dataset, or formal collaborative initiative for AI-assisted orthodontic material design currently exists. This absence is structurally significant. In computational materials science, open repositories—the Materials Project (>150,000 compounds), AFLOW (>1.8 million entries), and NOMAD—have become the enabling infrastructure for ML-driven property prediction and generative alloy design across battery, thermoelectric, and superalloy domains. None contain orthodontic-relevant experimental mechanical data, because stress relaxation profiles, superelastic plateau forces, and intraoral degradation kinetics are experimentally measured quantities that no community effort has yet organized to collect and share. In diagnostic orthodontic AI, the benchmark infrastructure analogy has been established: cephalometric datasets such as Cepha29 enabled systematic landmark detection benchmarking through deliberate collaborative effort. No equivalent exists for material performance. The data schema specified below is intended as a foundational specification for such an initiative; the federated learning architecture discussed in Section 7 provides a mechanism by which proprietary manufacturer data could contribute to shared models without requiring centralized disclosure.

A minimum viable standardized dataset for orthodontic materials AI must incorporate five domain-specific data layers, each with defined measurement conditions and reporting fields:

(1) Stress–strain mechanical behavior. For thermoplastic aligner polymers: elastic modulus, yield stress, ultimate tensile strength, and elongation at break from uniaxial tensile testing per ISO 527 [33], conducted at 37 °C in aqueous medium to approximate intraoral temperature. For archwire alloys: three-point bending load-deflection curves per ISO 15841 [34], including superelastic plateau force, activation and deactivation force values, and hysteresis area. These must be recorded at clinically relevant deflection ranges (0–3 mm) and temperatures (25 °C, 37 °C) since NiTi transformation behavior is temperature-sensitive within the oral range. Specimen geometry, manufacturer, batch number, and thermoforming or heat-treatment parameters must be co-recorded as covariates, as these are primary determinants of lot-to-lot variability.
(2) Viscoelastic response and time-dependent behavior. Stress relaxation profiles—recording force as a function of time at fixed deflection over clinically relevant wear periods (2, 4, 8, 24, 48, 168 h)—are essential for ML models predicting in vivo force decay in aligner polymers. Dynamic mechanical analysis (DMA) providing storage modulus, loss modulus, and tan δ as a function of frequency and temperature characterizes the frequency-dependent stiffness that governs force delivery under intermittent occlusal loading. These viscoelastic parameters are rarely reported in the current orthodontic materials literature, yet they are the mechanistically correct descriptors of aligner force delivery in function—static elastic modulus measured once at baseline does not capture the clinically relevant time course of force decay.
(3) Intraoral aging and degradation data. Mechanical properties must be recorded at multiple time points following accelerated aging protocols: immersion in artificial saliva at 37 °C (baseline, 24 h, 7 d, 14 d, 21 d), thermocycling (500–1000 cycles, 5–55 °C), and exposure to pH-cycled media simulating dietary acid challenge. Surface characterization—Ra surface roughness, contact angle, and XPS or FTIR spectroscopic analysis of surface chemistry—must accompany mechanical data, as surface degradation modulates both frictional behavior and biofilm accumulation. Ion release data (ICP-OES or ICP-MS for Ni, Ti, Cr, Co from metallic devices) must be included for toxicological completeness and regulatory compliance. Degradation data are currently the most severely underrepresented domain in the published literature: the majority of studies report only baseline properties, providing no training signal for ML models predicting clinical performance over the full treatment duration.
(4) Patient-specific loading conditions. In vivo force magnitudes, directions, and temporal profiles constitute the loading boundary conditions that determine how a material performs in a given patient. These are currently unmeasured in clinical practice. Instrumented aligner systems—such as the piezoelectric sensor platform developed by Feng et al. [16]—provide a mechanism for capturing patient-specific occlusal force data in real time. A standardized dataset must link material mechanical parameters to the loading environment in which they were measured: at minimum, bite force magnitude, contact area, and loading frequency should be co-recorded with material performance outcomes. Without this linkage, ML models trained on laboratory mechanical data cannot be validated against clinical force delivery, and the translation from in vitro property prediction to in vivo clinical outcome remains unresolved.
(5) Biological response parameters. Material performance is not defined solely by mechanical behavior but by the tissue response it produces. A complete dataset must therefore include the following: orthodontic tooth movement rate (mm/month, by arch segment) under defined force delivery conditions; OIRR occurrence and severity graded from CBCT at defined treatment intervals; clinical periodontal indices (bleeding on probing, probing depth) as indicators of soft tissue response to material surface exposure; and, where biomarker collection is feasible, gingival crevicular fluid (GCF) cytokine profiles (IL-1β, TNF-α, RANKL/OPG ratio) as proxies for the bone remodeling response to material-generated forces. These biological outcome variables transform a materials database into a materials-to-outcome database—the data structure required for ML models that predicts patient-specific biological response from material parameters, which is the clinical translation goal of this entire research agenda.

Harmonization of these five data layers across institutions requires consensus on measurement protocols—specifically, temperature and medium conditions for mechanical testing, aging protocol standardization, and minimum follow-up intervals for clinical outcome recording. The development of a reporting standard for orthodontic materials AI studies, modeled on MI-CLAIM for clinical AI, is a prerequisite for inter-institutional data pooling and the meta-analytic training datasets that high-performing ML models require.

### 6.2. Interpretability of Materials AI

Neural networks predicting material properties from composition or processing parameters offer limited mechanistic interpretability. For regulatory approval of AI-designed patient-contact devices, interpretability is not optional: regulators require understanding of why a material was selected, not merely that an algorithm recommended it. Explainable AI (XAI) methods—including gradient-weighted class activation mapping (Grad-CAM) and SHapley Additive exPlanations (SHAP) values—have been applied to clinical diagnostic AI in orthodontics [12], but their application to materials property prediction in dental contexts remains nascent. Interpretability frameworks for materials AI must be developed in parallel with predictive model building.

### 6.3. Handling Clinical Variability and Patient-Specific Biological Responses

Clinical variability in orthodontic treatment operates at three levels that AI frameworks must address explicitly: inter-patient biological variability (differences in PDL viscoelasticity, alveolar bone density and cortical thickness, root morphology, and individual remodeling rate); intra-patient temporal variability (changes in tissue properties over the treatment course, including PDL adaptation, bone remodeling, and aligner polymer degradation); and behavioral variability (wear time compliance, dietary habits, and oral hygiene, all of which alter the mechanical environment in which material properties are expressed). Each level requires a distinct methodological response.

Inter-patient biological variability is the most tractable of the three, because its primary drivers are measurable. PDL elastic modulus varies approximately 10-fold across individuals (0.05–0.68 MPa depending on loading direction and hydration state), alveolar bone density spans a clinically meaningful range quantifiable from CBCT Hounsfield units, and root morphology—particularly apical cross-sectional area and curvature—is directly segmentable from CBCT using validated AI tools. The appropriate ML framework for this level of variability is patient-stratified modeling: rather than training a single population-level model, ML models are trained on stratified subgroups defined by measurable anatomical and biological covariates, or the biological parameters are encoded directly as continuous input features in a regression or neural network architecture. The FEA-surrogate approach described in Section 5 is directly suited to this: a neural network trained on parametric FEA simulations that span the clinically observed range of PDL modulus, bone density, and root morphology encodes inter-patient variability implicitly in its input space, enabling patient-specific force and stress predictions without requiring a new simulation for each patient.

Intra-patient temporal variability—the fact that tissue mechanical properties change as treatment progresses and that aligner polymer properties degrade over the wear period—requires dynamic rather than static modeling. Here, recurrent neural network (RNN) architectures and Gaussian process (GP) time-series models are methodologically appropriate: they can update force delivery and tissue response predictions as new observational data become available (intraoral scan comparisons at appointment intervals, remote monitoring force data, or sequential CBCT measurements). Bayesian updating frameworks are particularly well suited to this problem because they provide calibrated uncertainty estimates that narrow as patient-specific data accumulate—beginning with population-level priors at treatment onset and converging toward individualized predictions as clinical observations are incorporated. This approach has been demonstrated in orthopedic implant load monitoring and is directly translatable to orthodontic material performance tracking.

Behavioral variability—compliance, diet, and oral hygiene—represents the least tractable source of uncertainty because it is not directly measurable from tissue or material properties. However, remote monitoring systems and instrumented aligners (as discussed in Section 2) are beginning to generate wear-time and force-profile data that can serve as proxies for compliance behavior. ML models trained on these behavioral signals could identify non-compliant wear patterns early and trigger clinical alerts or protocol adjustments before material force delivery diverges from the prescribed treatment plan. This converts compliance variability from an unobserved confounder into a monitored, manageable input variable.

A unifying architectural strategy for all three levels is the probabilistic prediction framework: rather than producing point estimates of material performance or tissue response, AI models should output calibrated probability distributions over predicted outcomes. Conformal prediction methods and Bayesian neural networks can provide prediction intervals that communicate the degree of uncertainty attributable to biological variability explicitly—enabling the clinician to make an informed decision about acceptable risk rather than receiving a single deterministic recommendation that obscures the inter-individual uncertainty on which it is conditioned. This is not merely a statistical nicety; for regulatory acceptance of AI-driven material decisions, demonstrated calibration of uncertainty is likely to be a prerequisite.

### 6.4. Regulatory Gap for AI-Designed Dental Devices

Current FDA and EU MDR frameworks for AI as Software as a Medical Device (SaMD) were designed around diagnostic and clinical decision-support tools. AI systems that determine material composition, alloy selection, or print parameters for a patient-contact orthodontic device occupy a distinct regulatory category—closer to AI-assisted manufacturing than to clinical decision support—for which no clear approval pathway has been established. This regulatory ambiguity will delay clinical translation of AI-driven orthodontic material optimization regardless of scientific maturity. The validation pathway for AI-designed orthodontic materials must proceed through four sequential stages before clinical translation is defensible. First, in vitro material validation: AI-optimized compositions or geometries must demonstrate mechanical performance—stress relaxation, superelastic plateau force, surface roughness, and degradation resistance—within or exceeding the range of clinically accepted comparators under standardized ISO testing conditions. Second, in vitro biological safety: cytotoxicity, ion release, and biocompatibility must be established per ISO 10993 [35] before any patient contact, regardless of the computational method used to design the material. Third, in vivo proof-of-concept: animal or early-phase human studies must confirm that AI-predicted force delivery and tissue response correspond to measured outcomes—tooth movement rate, OIRR incidence, PDL histology where available—establishing that the model’s predictions generalize beyond the training data distribution. Fourth, prospective controlled clinical trials: randomized comparison against current standard-of-care materials, with pre-registered primary endpoints (treatment duration, root resorption incidence, patient-reported outcomes), is required before claims of clinical superiority can be made and before regulatory submission under FDA SaMD guidance or European MDR Article 61 is viable. Technology readiness level (TRL) framing is useful here: most AI-driven orthodontic material concepts currently sit at TRL 2–3 (concept formulated, experimental proof of concept); clinical translation requires reaching TRL 7–8 (system prototype demonstrated in operational environment). Closing that gap is a decade-scale research commitment, not an incremental refinement.

### 6.5. Cost, Accessibility, and Global Equity

AI-driven material optimization, patient-specific digital twins, and federated learning infrastructure require computational, financial, and institutional resources that are unevenly distributed across the global orthodontic community. CBCT-based AI segmentation tools, FEA-surrogate modeling pipelines, and the data engineering staff required to maintain them are concentrated in well-resourced academic centers and large private clinics, predominantly in high-income countries. The orthodontic workforce in low- and middle-income countries—where the global burden of untreated malocclusion is highest—is unlikely to benefit from these tools in the near term unless deployment models account for resource asymmetry. Cloud-based inference services, federated learning protocols that do not require local high-performance computing, and tiered tool deployment (lightweight diagnostic support before resource-intensive material optimization) are potential mitigation strategies, but each raises its own data sovereignty and access-equity questions. The same regulatory pathways that protect patients in well-resourced jurisdictions can also delay or block access in jurisdictions with less developed device regulation. The AI–materials integration agenda described in this perspective should not be pursued in isolation from these considerations: a technology that improves outcomes only for patients in already-privileged settings would deepen rather than reduce orthodontic care inequality.

### 6.6. The Clinical Translation Gap

Even where AI material optimization tools exist or are developed, clinical uptake requires orthodontists to trust algorithmic material selection over established empirical habits. The evidence base for AI-driven material decisions in orthodontics is far thinner than for AI-driven imaging tasks, and clinician familiarity with materials science concepts is variable. This translation gap is not merely a technical problem—it requires interdisciplinary education, clinical validation trials, and professional society engagement to close.

## 7. Future Directions: The Authors’ Perspective

Three positions are advanced here as explicit, defended recommendations rather than neutral forecasts.

Position 1: AI-optimized polymer design will surpass diagnostic AI refinement as the highest-yield frontier within the next decade. CNN-based landmark detection and skeletal classification have achieved clinically acceptable accuracy; marginal improvements offer diminishing returns. AI-driven generative design of aligner polymers—simultaneously optimizing force delivery, stress relaxation, biocompatibility, and optical transparency for a given patient’s biomechanical profile—operates at TRL 2–3 and offers transformative improvement in treatment predictability. Research investment should shift accordingly.

Position 2: Digital twins integrating material properties with patient-specific anatomy are the necessary infrastructure for next-generation treatment planning. The material parameter layer includes (1) aligner polymer mechanical properties—elastic modulus, yield strength, and viscoelastic stress relaxation constants (characterizable from dynamic mechanical analysis of the specific polymer grade and sheet thickness used); (2) archwire alloy superelastic plateau force and transformation temperature range (measurable by three-point bending tests per ISO 15841); (3) bracket slot geometry and surface roughness (Ra), which together with wire modulus determine frictional resistance at the bracket–wire interface; and (4) for additively manufactured devices, print-parameter-dependent modulus and surface roughness as predictable from the supervised ML models discussed in Section 4.

The mechanical parameter layer includes (1) PDL elastic modulus and viscoelastic time constants—values spanning approximately 0.05–0.68 MPa depending on loading direction and rate, derivable from micro-CT-based inverse FEA or increasingly from AI-assisted CBCT tissue characterization; (2) alveolar bone density and cortical thickness, extractable from CBCT using validated Hounsfield unit calibration; (3) root morphology descriptors—length, curvature, cross-sectional area at the apical third—quantifiable from CBCT-based AI segmentation; and (4) tooth-specific periodontal space width, which modulates force transmission efficiency.

The biological parameter layer includes (1) patient-specific OIRR susceptibility indices derived from root morphology classification (pipette-shaped roots conferring elevated risk) and treatment duration projections; (2) bone remodeling rate proxies, potentially derivable from sequential CBCT volumetric measurements at defined intervals; and (3) genetic or inflammatory markers of resorption susceptibility where available, though integration of biomarker data into clinical DT frameworks remains at concept stage.

This parameter set is not speculative: each entry is either currently measurable in a clinical or laboratory workflow or is within one developmental step of clinical measurability given existing AI segmentation and material characterization methods. The barrier to digital twin implementation is not parameter availability per se but the absence of a data integration architecture that connects material testing outputs, CBCT-derived anatomical measurements, and in vivo force monitoring into a unified patient record that an ML surrogate model can query at the point of treatment planning. This is technically feasible; the barrier is interdisciplinary data integration and the organizational infrastructure to support it.

Position 3: Federated learning is the mechanism by which the orthodontic materials community can build the training datasets the field requires. Individual clinics and manufacturers cannot generate sufficient material-performance data alone. Federated learning—training models across distributed datasets without centralizing proprietary data—has demonstrated feasibility in orthodontic AI contexts [36] and is directly applicable to material performance prediction across clinics, manufacturers, and research institutions.

Closing the AI–materials gap requires not only research investment but a defined collaboration architecture. We propose a four-role framework organized around a shared material-performance dataset as the central integrating object. Orthodontists define clinically meaningful performance endpoints—minimum force thresholds, acceptable force decay rates, OIRR risk tolerance—and contribute longitudinal patient outcome data; they are the domain authority on what the material must achieve biologically and clinically. Materials scientists design and execute standardized in vitro characterization protocols (ISO 527, ISO 15841, ISO 10993), curate the resulting datasets, and validate AI-predicted properties against physical measurements. Computational researchers build and maintain FEA-surrogate and digital twin models, define the data schema, and ensure dataset interoperability across institutions via federated learning infrastructure. AI specialists develop, audit, and interpret the ML models—managing training pipelines, uncertainty quantification, and XAI outputs required for regulatory submission. The critical interface between these roles is a standing data governance agreement specifying measurement protocols, data fields, update cadence, and intellectual property terms—without which interdisciplinary collaboration defaults to parallel activity rather than integrated research. Existing precedents in oncology (TCGA) and genomics (GA4GH) demonstrate that such governance frameworks are achievable; the orthodontic biomaterials community requires an equivalent.

The literature gaps documented across Section 2, Section 3, Section 4 and Section 5 translate directly into a prioritized research agenda. For the next 1–3 years, the field’s most tractable contributions are: standardized in vitro characterization datasets for PETG and TPU aligner polymers under ISO 527 conditions with stress relaxation profiling (Section 2); Bayesian optimization studies of NiTi ternary alloy compositions targeting orthodontic-specific force and temperature windows (Section 3); and prospective validation of supervised ML print-parameter models across multiple DLP resin chemistries and printer platforms (Section 4). For the 3–7-year horizon, the priority is FEA-surrogate model external validation across multi-institutional patient cohorts with diverse skeletal morphologies (Section 5) and first-in-human pilot studies linking AI-predicted force delivery to measured tooth movement and OIRR outcomes. Beyond 7 years, the defining challenge is regulatory acceptance of AI-assisted material selection as a clinical decision support tool—a process that will require the standardized datasets, validation hierarchy, and interdisciplinary governance framework outlined in Section 6 and Section 7 to be in place. These timelines are estimates, not guarantees; the rate-limiting step in each case is data infrastructure, not algorithmic capability.

**Figure 2:**
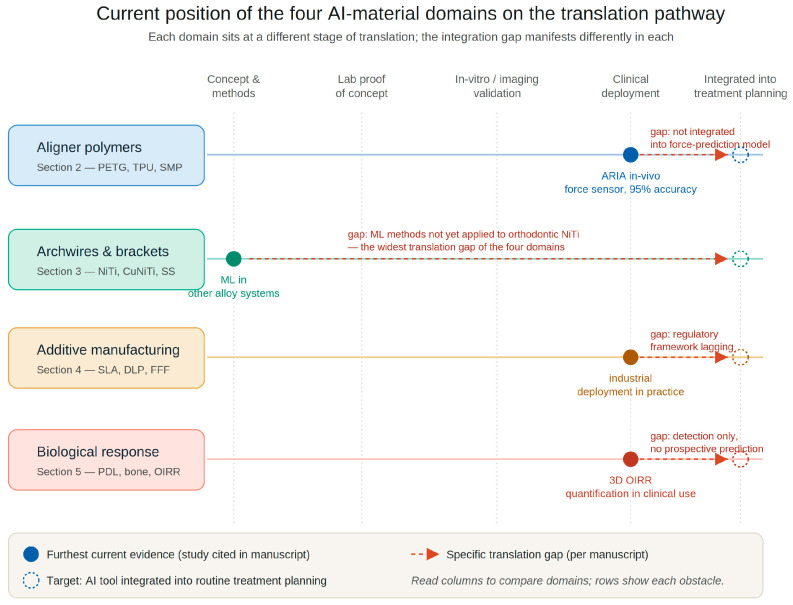
Current position of the four AI–material domains examined in this perspective on a five-stage translation pathway. For each domain (aligner polymers, archwires and brackets, additive manufacturing, biological response), the filled colored circle indicates the furthest current evidence position, anchored to a specific study cited in the manuscript. The open dashed circle indicates the target state of integration into routine orthodontic treatment planning. The dashed red arrow indicates the specific translation gap, labeled with the principal obstacle identified in the corresponding section of the manuscript. The non-uniform distribution of current positions across domains illustrates that the AI–materials integration gap manifests differently in each domain: as absence of methods in archwires, absence of integration in aligner polymers, regulatory lag in additive manufacturing, and absence of prospective application in biological response prediction. OIRR = orthodontically induced root resorption; PDL = periodontal ligament; SS = stainless steel; SLA = stereolithography; DLP = digital light processing; FFF = fused filament fabrication; SMP = shape memory polymer; PETG = polyethylene terephthalate glycol; TPU = thermoplastic polyurethane; ML = machine learning [16,18,23,26].

Figure 2 synthesizes the current state of the four domains examined in this perspective against a unified translation pathway. The positioning of each domain reflects the furthest published evidence cited in the corresponding section: aligner polymers have advanced to clinical deployment through in vivo force sensing [16], additive manufacturing through industrial deployment of ML-optimized printing parameters [23], and biological response prediction through clinically deployed deep-learning OIRR quantification [26]. Archwires and brackets lag significantly behind, with the ML methods relevant to alloy design demonstrated only in non-orthodontic systems [18]. The figure makes visible a central observation of this perspective: the AI–materials integration gap is not uniform across orthodontic material domains, and the specific obstacle preventing each domain from reaching clinical integration differs. Research prioritization, regulatory dialogue, and resource allocation should accordingly be domain-specific rather than treating AI in orthodontic materials as a single research programme.

## 8. Conclusions

This perspective identifies and characterizes a structural gap in digital orthodontics: AI-optimized treatment plans are executed through empirically selected materials whose mechanical behavior the planning system never models. Across four domains—thermoplastic aligner polymers, NiTi archwire alloys, additive manufacturing, and biological response prediction—we demonstrate that AI tools capable of closing this gap exist but are not yet connected to clinical workflows. The novel contribution is the first systematic mapping of AI applications along the materials axis rather than the diagnostic axis of orthodontic practice. The practical implication is specific: force delivery, material degradation, and patient-specific tissue response are all ML-tractable problems given appropriate datasets. Closing the gap requires standardized open material-performance repositories, FEA-surrogate models integrating polymer and alloy properties as treatment-planning inputs, patient-specific digital twins, and a four-role interdisciplinary governance framework. The next decade’s defining challenge is not algorithmic—it is infrastructural.

## Figures and Tables

**Table 1:** Comparative overview of AI applications, identified gaps, and clinical relevance across the four orthodontic material domains examined in this perspective. All entries are summarized directly from Section 2, Section 3, Section 4 and Section 5 of the manuscript without addition of external claims.

Domain	Current AI Application Reported in This Perspective	Gap Explicitly Identified in This Perspective	Clinical Relevance Stated in This Perspective	Section
Thermoplastic aligner polymers	Neural networks predict thermoforming-dependent mechanical properties from temperature, sheet thickness, and cooling rate; embedded piezoelectric sensor aligners generate force data (ARIA system, 95% classification accuracy across >1400 datasets).	No AI system integrates polymer mechanical properties into force prediction. Material choice (PETG vs. TPU) in clinical practice is made empirically, without quantitative modeling of polymer–patient interaction.	PETG vs. TPU selection determines whether bodily retraction achieves root displacement or crown tipping; the authors report differing clinical outcomes between polymer types for identical virtual setups.	Section 2
Archwire and bracket alloys	ML approaches across alloy systems demonstrate adaptive design strategies combining ML predictions with targeted experiments to accelerate alloy composition identification with specified transformation behavior.	These methods have not yet been systematically applied to orthodontic wire alloy optimization. ML-based bracket-wire friction prediction from multi-parameter datasets is proposed but not implemented.	NiTi superelastic plateau force is sensitive to alloy composition, wire diameter, heat treatment, and oral temperature, making in vivo force prediction from standard in vitro tests unreliable.	Section 3
Additive manufacturing of orthodontic devices	Artificial neural networks and support vector regression predict surface roughness of 3D-printed dental resins from five input parameters (layer thickness, infill density, print angle, exposure time, lift speed). Closed-loop AI quality control reduces waste up to 90%.	Long-term mechanical performance data under oral conditions are substantially thinner than for thermoformed thermoplastics. Biocompatibility data sufficient for ML training are fragmented and largely proprietary. No regulatory framework for AI-process-optimized patient-contact devices exists.	Surface roughness affects plaque accumulation on aligners/retainers (caries and periodontal risk) and bracket-adhesive bond strength.	Section 4
Biological response prediction	Deep learning models perform 3D OIRR quantification from CBCT (5–7 h reduced to 20–30 min); 100% specificity reported for apical-third resorption. FEA-surrogate feedforward networks demonstrated in a 2025 implant-loading proof of concept.	Existing tools detect damage retrospectively. No system predicts, prior to treatment, which patients are at elevated risk given their specific PDL material properties and the forces a given aligner polymer or archwire will generate.	OIRR affects 40–60% of orthodontic patients and is the most significant irreversible adverse effect of treatment; force level—a direct function of the material used—is independently associated with OIRR severity.	Section 5

ARIA = aligner system with embedded piezoelectric sensors [16]; CBCT = cone-beam computed tomography; FEA = finite element analysis; ML = machine learning; NiTi = nickel-titanium; OIRR = orthodontically induced root resorption; PDL = periodontal ligament; PETG = polyethylene terephthalate glycol; TPU = thermoplastic polyurethane.

## Data Availability

The original contributions presented in the study are included in the article, further inquiries can be directed to the corresponding author.

## Abbreviations

The following abbreviations are used in this manuscript: AI—Artificial Intelligence; ANN—Artificial Neural Network; CAT—Clear Aligner Therapy; CBCT—Cone-Beam Computed Tomography; CNN—Convolutional Neural Network; DLP—Digital Light Processing; EU MDR—European Union Medical Device Regulation; FEA—Finite Element Analysis; FDA—U.S. Food and Drug Administration; IOS—Intraoral Scan; ML—Machine Learning; NiTi—Nickel-Titanium; OIRR—Orthodontically Induced Root Resorption; PDL—Periodontal Ligament; PETG—Polyethylene Terephthalate Glycol; QC—Quality Control; Ra—Arithmetic Mean Surface Roughness; SaMD—Software as a Medical Device; SHAP—SHapley Additive exPlanations; SLA—Stereolithography; SMP—Shape Memory Polymer; SVR—Support Vector Regression; TPU—Thermoplastic Polyurethane; TRL—Technology Readiness Level; XAI—Explainable Artificial Intelligence.
